# Analyses of amplified fragment length polymorphisms (AFLP) indicate rapid radiation of *Diospyros* species (Ebenaceae) endemic to New Caledonia

**DOI:** 10.1186/1471-2148-13-269

**Published:** 2013-12-12

**Authors:** Barbara Turner, Ovidiu Paun, Jérôme Munzinger, Sutee Duangjai, Mark W Chase, Rosabelle Samuel

**Affiliations:** 1 Department of Systematic and Evolutionary Botany, Faculty of Life Sciences, University Vienna, Rennweg 14, 1030 Wien, Austria; 2 IRD, UMR AMAP, TA A51/PS2, 34398 Montpellier Cedex 5, France; 3 Department of Forest Biology, Faculty of Forestry, Kasetsart University, Bangkok, Thailand; 4 Jodrell Laboratory, Royal Botanic Gardens, Kew, Richmond, Surrey TW9 3DS, UK; 5 School of Plant Biology, The University of Western Australia, Crawley, WA 6009, Australia

**Keywords:** Cryptic species, Island flora, Morphological diversification, Progenitor/derivative relationships, Species radiation, Woody plants

## Abstract

**Background:**

Radiation in some plant groups has occurred on islands and due to the characteristic rapid pace of phenotypic evolution, standard molecular markers often provide insufficient variation for phylogenetic reconstruction. To resolve relationships within a clade of 21 closely related New Caledonian *Diospyros* species and evaluate species boundaries we analysed genome-wide DNA variation via amplified fragment length polymorphisms (AFLP).

**Results:**

A neighbour-joining (NJ) dendrogram based on Dice distances shows all species except *D. minimifolia*, *D. parviflora* and *D. vieillardii* to form unique clusters of genetically similar accessions. However, there was little variation between these species clusters, resulting in unresolved species relationships and a star-like general NJ topology. Correspondingly, analyses of molecular variance showed more variation within species than between them. A Bayesian analysis with BEAST produced a similar result. Another Bayesian method, this time a clustering method, Structure, demonstrated the presence of two groups, highly congruent with those observed in a principal coordinate analysis (PCO). Molecular divergence between the two groups is low and does not correspond to any hypothesised taxonomic, ecological or geographical patterns.

**Conclusions:**

We hypothesise that such a pattern could have been produced by rapid and complex evolution involving a widespread progenitor for which an initial split into two groups was followed by subsequent fragmentation into many diverging populations, which was followed by range expansion of then divergent entities. Overall, this process resulted in an opportunistic pattern of phenotypic diversification. The time since divergence was probably insufficient for some species to become genetically well-differentiated, resulting in progenitor/derivative relationships being exhibited in a few cases. In other cases, our analyses may have revealed evidence for the existence of cryptic species, for which more study of morphology and ecology are now required.

## Background

Island floras are often characterized by high levels of endemism and groups of closely related but morphological and ecological divergent species that are mostly the result of single colonisation events followed by radiation e.g. [1,2]. New Caledonia was cited as one of the 34 biodiversity hotspots recognized by Conservation International [3,4]. Nearly 75% of the native flora is endemic [5], which is the fourth highest for an island [6]. While the continental part of New Caledonia (mainly Grande Terre) was entirely submerged during the Eocene (until 37 mya), a thick layer of heavy-metal-rich oceanic mantle accumulated [7]. Today, around one-third of the main island, Grande Terre, is still overlaid with ultramafic substrates. Generally, Grande Terre is a substrate mosaic [8], which is cited as one reason for the high level of endemism found there e.g. [9]. The climate in New Caledonia ranges from tropical to subtropical, and the main island is split by a mountain range into a humid eastern and a dry western part with prevailing winds and rain coming from the south-east. Taking climatic and geological factors together, Grande Terre has a wide range of environmental diversity. The main vegetation types in New Caledonia are evergreen humid forests, maquis, dry forests, littoral vegetation, and savannah [10].

One plant group that has taken advantage of many available habitats on New Caledonia is *Diospyros*, which is the largest genus (> 500 species in its broad circumscription [11]) of Ebenaceae, a pantropical family of woody plants. In New Caledonia *Diospyros* species range from sea level up to ca. 1250 m (the highest point New Caledonia is 1628 m), and species are found in all vegetation types except mangroves, with several species co-occurring in micro-sympatry (Table 1).

**Table 1:** **Occurrence of *Diospyros *** **species in different habitats in New Caledonia**

		**Substrate**				
		**Limestone**	**Serpentine**	**Schist**	**Ultramafic rock**	**Volcanic rock**
Vegetation	Humid mountain forest			*D. parviflora*, *D. trisulca*		*D. flavocarpa*, *D. labillardierei*
	Humid low land forest				*D. glans*, *D. pancheri*, *D. parviflora*, *D. umbrosa*	*D. umbrosa*
	Mesophyll forest	*D. minimifolia*, *D. pustuala*, *D. tridentata*			*D. erudita*	*D. cherrieri*, *D. erudita*, *D. minimifolia*, *D. perplexa*, *D. pustulata*
	Maquis		*D. revolutissima*		*D. vieillardii*	
	Littoral forest	*D. calciphila*, *D. inexplorata*				*D. impolita*

Habitats are grouped according to vegetation type and substrate. Note that several species are co-occurring and that a few species are found in several habitats.

*Diospyros* colonised New Caledonia via long-distance dispersal at least four times [12]. In previous studies based on low-copy nuclear and/or multiple plastid markers [12,13], it was possible to resolve phylogenetic relationships for the majority of *Diospyros* species, except for one group of endemics from New Caledonia. Of the 31 New Caledonian *Diospyros* species, 24 belong to this clade of closely related endemics. In previous analyses, this strongly supported group is related to species found on islands throughout the Indian and Pacific Oceans as far east as Hawai’i [12,13]. However, due to extremely low levels of sequence divergence, it was not possible to tease apart relationships between these species (they formed a hard polytomy in most individual trees, and there was little informative variation that permitted clustering of pairs or groups of species). Most of these closely related species are morphologically and ecologically clearly differentiated (for examples see [13]), and several species are narrow endemics restricted to small areas.

Amplified fragment length polymorphism (AFLP; [14]) is a fingerprinting technique that has proven to be useful for revealing phylogenetic relationship among closely related taxa (e.g. *Hypochaeris*, [15]; *Lactuca*, [16]; *Phylica*, [17]; *Trollius*, [18]; *Ranunculus alpestris*, [19]; *Puya*, [20,21]; *Araucaria*, [22]). In contrast to standard phylogenetic markers, AFLP variation is spread across the whole genome, spanning both coding and non-coding DNA regions and may therefore be more representative of overall genetic patterns present as well as being highly informative for phylogenetic analyses at the low phylogenetic level [23,24]. Compared to other fingerprinting techniques AFLP shows increased reproducibility and does not require any prior knowledge of the analysed genomes. However, there are some detrimental issues to consider when working with AFLP data; these include potential non-homology and non-independence of fragments, asymmetry in the probability of loss/gain of fragments, and problems in distinguishing heterozygote from homozygote bands e.g. [23,25]. Despite these difficulties, several authors have used AFLPs to reveal phylogenetic relationships corroborated by analyses of other types of data, especially for species that have diverged recently or radiated within a short period of time e.g. [15,17,23,26].

**Figure 1:**
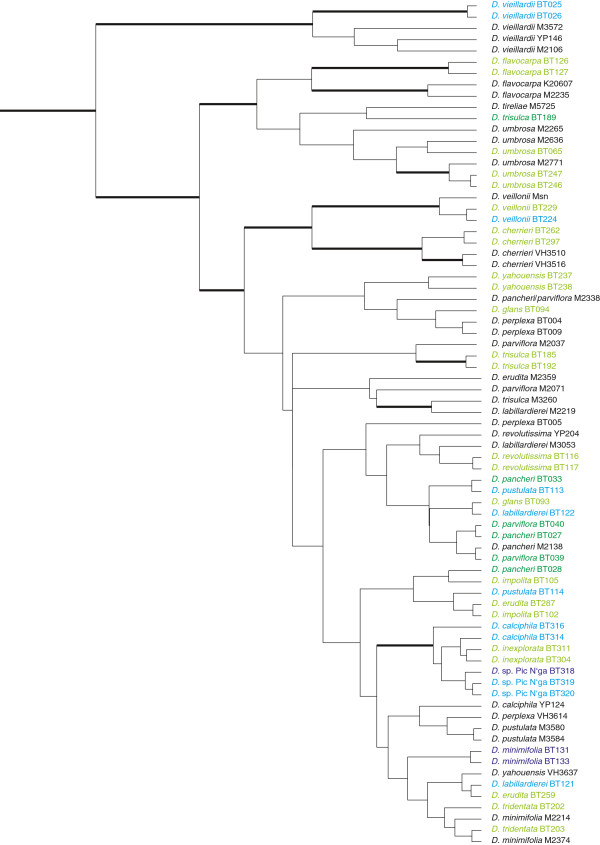
**Bayesian maximum clade credibility tree of New Caledonian *Diospyros *species based on plastid and nuclear DNA data (taken from Turner et al.**[13]**).** Bold branches are supported (> 70% bootstrap and Bayesian posterior probability). Accessions in blue correspond to the white group found in Structure and PCO, green ones to the grey group (light blue/green accessions included in current data set, dark blue/green accessions failed in current analysis but colour indicates the group to which they most probably belong), accessions in black are not included in the present study.

In this study we focus on this group of closely related species of *Diospyros* endemic to New Caledonia (Figure 1). Our aim was to clarify species boundaries as well as phylogenetic relationships between these New Caledonian *Diospyros* species. Integrated in a broader context, the outcome of our research should help us better understand the factors behind and mechanisms of speciation and radiation on islands.

## Results

After excluding 186 replicates the final matrix used for analyses contained 192 individuals and 792 fragments. The AFLP profiles showed good reproducibility with a mean error-rate of 2.4% across all replicated samples. Because the focus of this study was on the phylogenetic relationships between species and species limits rather than intra-specific population genetics, we are presenting and discussing mostly the results of inter-specific relationships. We are presenting here only unrooted trees due to the low resolution of their backbone. We analysed the data using neighbour-joining (NJ) dendrograms and principal coordinate analysis (PCO) with different distance methods, and in both cases the Dice distance gave the highest resolution of relationships between species.

**Figure 2:**
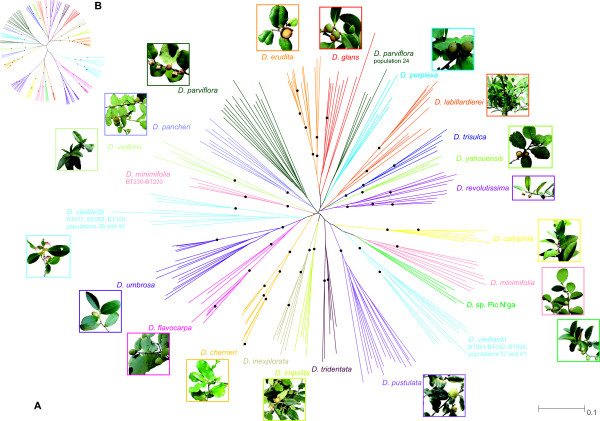
**Phylogenetic dendrograms inferred from the data collected in this study.** Each species is shown in a different colour. Colours were selected randomly and do not indicate any grouping. **A**: Neighbour joining dendrogram based on Dice distances. Black dots indicate nodes with > 80% bootstrap support. **B**: Bayesian maximum clade credibility dendrogram. Black dots indicate nodes with > 0.95 Bayesian posterior probability. Picture credits: *D. calciphila*: H. Benoît, http://www.endemia.nc; *D. cherrieri*: C. Chambrey; *D. erudita*, *D. pancheri*, *D. pustulata*, *D. umbrosa*, *D. vieillardii*: D. & I. Létocart, http://www.endemia.nc; *D. flavocarpa*, *D. minimifolia*, *D. revolutissima*, *D.* sp. Pic N’ga: J. Munzinger; *D. glans*, *D. parviflora*: J.-L. Ruiz, http://www.endemia.nc; *D. impolita*: J. Barrault, http://www.endemia.nc; *D. labillardierei*: B. Turner; *D. perplexa*, *D. yahouensis*: V. Hequet; *D. veillonii*: R. Amice, http://www.endemia.nc.

The NJ analysis resulted in a star-like dendrogram with a backbone of short branches lacking bootstrap support greater than 75%. All species except *D. minimifolia*, *D. parviflora* and *D. vieillardii* form single clusters in the NJ tree (Figure 2A). However, only eight (*D. calciphila*, *D. cherrieri*, *D. inexplorata*, *D. impolita*, *D. pustulata*, *D. trisulca, D. umbrosa* and *D. yahouensis*) of the 21 included species form clusters with bootstrap higher than 80%. The Bayesian inference (BI) produces a similar result. All species except *D. labillardierei*, *D. minimifolia*, *D. pancheri* and *D. parviflora* form single clusters in the BI tree (Figure 2B). Apart from *D. flavocarpa*, *D. revolutissima*, *D. tridentata* and *D. vieillardii*, all clustered species have high (> 0.95) posterior probabilities.

**Figure 3:**
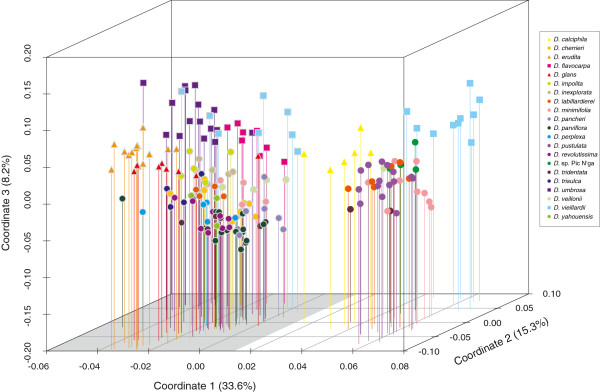
**PCO of individual accessions based on Dice distances.** Shading of the base-grid of the figure marks the two main groups inferred by Structure analysis – white and grey. Each species is shown in a different colour. Colours were selected randomly and do not indicate any grouping.

**Figure 4:**
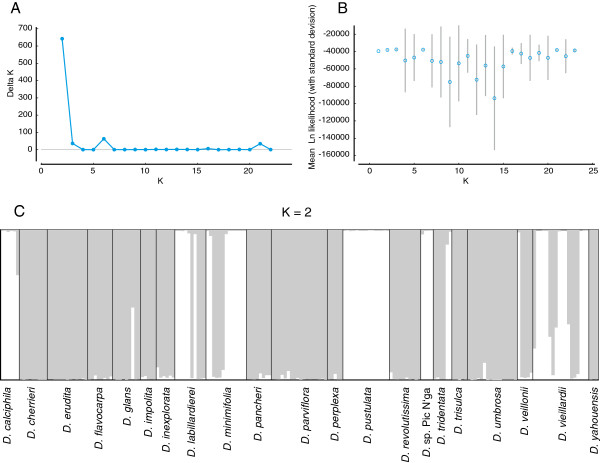
**Results of the Structure analysis. A)** Delta K values of the K values tested. **B)** Mean Ln likelihood of K values tested. **C)** Clustering of K = 2. The two groups are marked in different shades (white and grey).

PCO separated accessions into two main groups (hereafter named “white” and “grey”) that can be subdivided into six subgroups (Figure 3). Within the “white” group (defined in the Structure results below) subgroup one includes *D. vieillardii* (individuals indicated by squares in Figure 3), subgroup two *D. calciphila* (triangles) and subgroup three the rest of the individuals from this group (circles). In the “grey” group (more extensively described in the Structure results below) subgroup four included *D. flavocarpa*, *D. umbrosa* and *D. vieillardii* (indicated by squares in Figure 3), subgroup five *D. erudita* and *D. glans* (triangles) and subgroup six the remaining individuals (circles). A PCO of populations (not shown) based on the pair-wise *F*_*ST*_ distances obtained from the AMOVA resulted in similar groups and subgroups of populations as those obtained from the individual-based PCO. Structure analysis gave the highest value of ∆K for K = 2 plus few other suboptimal K values (Figure 4A and B). However the latter contained clusters with negligible membership (“empty” clusters). Both K = 3 and K = 6 resulted in three visible clusters, with one cluster being only found in significantly admixed samples (Additional file 1). Visualisation of K =16 and K = 21 showed two clusters only and both analyses are highly similar to each other (Additional file 1). It has been argued the ad-hoc Evanno method [27] favours by default K =2 over K = 1 when searching for the correct number of clusters [28]. However, PCO separated individuals included in our analyses into two groups as well, and therefore we consider K = 2 as representative for our sample set. For K = 2, the allele-frequency divergence between the two groups was 0.0074. One group (“grey”) includes the majority of accessions (Figure 4C). The other group (“white”) includes *D. calciphila*, *D. labillardierei* (population 13 and accession BT179), *D. minimifolia* (majority of individuals), *D. pustulata*, *D.* sp. Pic N’ga, *D. tridentata* (accessions BT206 and BT207), *D. veillonii* (accession BT224) and *D. vieillardii* (population 37 [except accession BT017], population 39 [except accession BT100] and population 41). Seven individuals appear to be admixed (less than 90% identity with one of the groups); most of those are *D. vieillardii*. Several species (*D. labillardierei*, *D. minimifolia*, *D. tridentata*, *D. veillonii* and *D. vieillardii*) and even some populations comprise individuals belonging to each of the two groups.

In order to quantify the amount of genetic variation between species we have performed a non-hierarchical AMOVA with species assigned as “populations”. This analysis showed as little as 30% of the variation to occur among the species. However, in the Structure, PCO, NJ and BI analyses several species seemed to be formed by genetically distinct populations assigned to different clusters and coming in distinct positions in the tree. To avoid mixing up of cryptic variation within a group, we run further AMOVAs with populations assigned as sample localities, despite the relatively low sample size per locality. Results of non-hierarchical AMOVA in this case indicate a higher level of differentiation between populations, resulting in an *F*_*ST*_ of 0.38. There was no visible difference in gene diversity between stands of co-occurring species and isolated populations. Several hierarchical AMOVAs (except one based on the Structure results) were not significantly more informative than the non-hierarchical AMOVAs (Table 2). Grouping populations according to geography or ecology, explains a surprisingly low amount of the variation (1.4 – 1.6%). Furthermore, allocating populations to the 21 included species assigns a relatively high percentage of variation at the between-species level (19.4%), but with a highly similar *F*_*ST*_ value to the non-hierarchical AMOVA results. When higher-level groupings paralleled the Structure results, we obtained the highest *F*_*ST*_ value (0.4), albeit the percentage of variation between the two clusters as defined by Structure was only 9.5%, lower than the percentage of differentiation shown between species. Removing seven admixed samples (less than 90% membership form each of the two groups based on the Structure results) from the AMOVA gave nearly the same results as the analysis including them (Table 2).

**Table 2:** Results of different AMOVAs conducted

	**Percentage of variation**				
**Analysis**	**Among groups**	**Among populations within groups**	**Within groups**	***F***_***ST***_	***p *** **value**
Non-hierarchical	-	38.16	61.84	0.382	0.00
Species-wise	19.43	19.15	61.42	0.386	0.00
Structure	9.46	33.22	57.32	0.427	0.00
Structure no admixed	9.93	33.39	56.68	0.433	0.00
Geographic	1.58	36.97	61.45	0.385	0.00
Water	1.37	37.20	61.43	0.386	0.00
Soil	1.54	36.92	61.54	0.385	0.00

In the non-hierarchical analysis, no grouping was applied. In the species-wise analysis, samples were grouped according to taxonomic features (21 groups corresponding to the 21 species included).

In the Structure analysis, samples were grouped according to the results of Structure analysis (two groups corresponding to the two groups – white and grey – inferred by Structure); in the analysis without admixed samples seven samples with less than 90% identity to one of the two groups were removed. In the geography analysis the samples were grouped according to their origin (three areas – north, middle and south – of New Caledonia). The analysis based on water availability was structured into two groups – humid and dry. In the soil-type based analysis, species were grouped according to the substrate on which they were found (five groups – limestone, serpentine, schist, ultramafic rock and volcanic rock).

The average gene diversity over loci within populations ranged from 0.03 in *D. erudita* (population 4) to 0.12 in *D. parviflora* (population 22). Contrary to predictions, the highest number of polymorphic sites, pair-wise differences and average gene diversity were not found in the admixed populations (according to the Structure results) but in *D. parviflora* (for details see Additional file 2).

## Discussion

“Explosive” radiations featuring rapid opportunistic morphological and ecological diversification are phenomena previously reported for some islands (e.g. [29] and references therein). Extreme ancestral bottlenecks, together with on-going hybridization and incomplete lineage sorting, can prevent phylogenetic reconstruction in cases of island radiations if they have been recent and produced many species [30]. However, a good understanding of phylogenetic relationships within radiating groups is key for further evolutionary studies into mechanisms and whether change is adaptive, due to drift in small populations or other phenomena [29].

For the endemic New Caledonian *Diospyros* species, previous studies, based on multiple plastid [12] and low-copy nuclear [13] markers, showed 21 species to be closely related (Figure 1) and were not able to clearly resolve phylogenetic relationships among them. In the combined data set (plastid and nuclear markers; [13]) only seven of the 21 species included were found to form highly supported groups of accessions from single species. Individuals belonging to each of the remaining 14 species failed to cluster according to their taxonomic circumscription. Dating analysis based on plastid and low-copy nuclear markers showed that the common ancestor of this clade of endemic New Caledonian *Diospyros* species has arrived in New Caledonia around nine million years ago [13]. *Diospyros vieillardii* has been shown to be sister to the rest of this endemic clade and separated from the rest of the species around 7.2 million years ago.

Results of the current study using genome-wide AFLP markers reveal that most species form unique groups paralleling recognised species. Around one-third (eight species, NJ dendrogram, Figure 2A) and one-half (11 species, Bayesian tree, Figure 2B) of the species, are genetically distinct with high support (Figure 2). However, the overall AFLP results prove unable to clearly resolve the backbone of trees, similar to previous results obtained from analyses of DNA sequence data [13]. Intra-specific variation was greater (~80%) than that found at inter-specific level (~20%). This low ratio of among- versus within-species divergence in the context of considerable morphological and ecological divergence is indicative of a recent diversification [22]. Such a process can explain why we were able to get clear species boundaries for most species but were unable to clearly resolve phylogenetic relationships between them.

Two species that did not form well-defined clades (*D. minimifolia* and *D. parviflora*) were previously considered by White [31] to show variability in leaf morphology that may indicate that they are in fact a collection of several species. For *D. minimifolia* White [31] mentioned that the type population (close to population 15 of this study) has smaller leaves compared to other populations of this species. In our results this population clusters together with the majority of the *D. minimifolia* accessions; the population that is separated from the rest (population 16) is from Gaji. According to White [31]*D. parviflora* is a wide-spread species, showing considerable variability of leaf morphology even within populations, making it impossible to differentiate these into different species. Our results show all accessions of *D. parviflora*, except those from Plateau de Tango (population 24), to form a group. All included accessions from *D. parviflora* are from ultramafic localities.

To our surprise, the AFLP results do not show any significant grouping according to ecological (edaphic, climatic, elevational), geographical or morphological factors (Additional file 3). The two weakly differentiated groups revealed by Structure and PCO also do not correspond to any conspicuous phenotypic characteristics. The allele-frequency divergence between the two groups found by Structure is low, which explains why we did not observe the two groups in the Bayesian and NJ tree-building results. Taken together, these results indicate that positive selection has perhaps acted on few genomic regions [32] and has resulted in phenotypic diversification of New Caledonian *Diospyros*. Variation in copy number of specific genomic regions may be an additional aspect of molecular variation that, although invisible to AFLP markers, could form the basis of adaptation to different environmental conditions [33].

The individuals of *D. vieillardii*, *D. umbrosa* and *D. flavocarpa* form a minimally isolated group (squares in the grey group) in the PCO (Figure 3). Previous phylogenetic analyses (Figure 1) showed these three species to be sister to the rest of the taxa. Due to its morphological and ecological features *D.* sp. Pic N’ga from Île des Pins could be a hybrid between *D. calciphila* and *D. vieillardii*, but *D. vieillardii* is now not known from this island. In PCO, individuals of this putative species are located between individuals of *D. calciphila* and *D. vieillardii* (Figure 3). The split between the two groups observed (Figures 3 and 4) could be relatively old, separating two lineages that developed in isolated regions. For instance, dry periods of the Pleistocene caused aridification in many areas, and some vegetation types persisted only in local refugia e.g. [34-36]. After climatic conditions became more favourable, the two groups probably expanded rapidly into newly suitable habitats where they overlapped; the time scale of these fluctuations (ca. 0.02 – 0.1 myr; [37]) was probably not enough to allow woody species with long generation time such as *Diospyros* to diverge and become permanently reproductively isolated [22]. There are a few admixed individuals in the Structure analysis (Figure 4), which implies that hybridization might have played a role in evolution of this group.

Accelerated rates of evolution at few genes as a result of positive selection could have resulted in the morphological and ecological diversification apparent today in this group of New Caledonian *Diospyros* species. Furthermore, in addition to retention of ancestral polymorphisms, frequent gene flow could have acted against genome-wide genetic differentiation between the species. Barriers to gene flow between these species may be highly porous, with only few genes responsible for ecological and morphological adaptations evolving on distinct trajectories under strong selection, which leaves the rest of their genomes open to gene flow [38]. Finding these few genes with AFLP is realistically improbable because they are a miniscule component in comparison the rest of these genomes. In the case of a recent and rapid radiation in plants, it could be argued that the bulk of regions sampled by AFLP have not evolved quickly enough to accumulate substitutions that could indicate species relationships. Our results are similar to those found in various other island genera (e.g. *Araucaria* in New Caledonia, [22]; *Ourisia* in New Zealand, [39]).

*Diospyros vieillardii*, which is sister to the rest of the taxa belonging to this group of New Caledonian endemics [12,13], is confined to ultramafic soils, which supports the hypothesis of this being an exaptation of the progenitor of this New Caledonian *Diospyros* clade to ultramafic soils when the whole island was still covered by heavy-metal-rich substrates; similar findings have been made in other plant groups in New Caledonia e.g. [9]. Later, erosion reduced the extent of this geological layer to one third of the island [7], and existing species began to move onto other substrates where they subsequently diverged, forming distinct species. Such observations have been made in various other New Caledonian groups (e.g. *Araucaria*, [22]; *Spiraeanthemum*, [35]; *Codia*, [40]). A few studies have examined the adaptive basis and processes involved in rapid radiations in New Caledonia e.g. [41] and Hawai’i (e.g. lobeliads, [42]; silverswords, [43]). Linking ecological parameters and/or phenotypic traits associated with speciation has to be done with caution because range alterations, subsequent evolution, and species extinctions might have erased initial signals found in only a few genes. Therefore, the associations observed today may be misleading, and the specific conditions/traits that were indeed linked to speciation, if any, may no longer be present [44].

Further work involving common garden experiments would provide insights into the effect of environmental conditions on morphological traits and therefore plasticity of genomes of the New Caledonian *Diospyros* species. Unfortunately, such experiments are time and cost intensive. It is difficult to obtain ripe fruits of all *Diospyros* species, and in addition it is difficult to germinate and grow them, which is a crucial aspect of conducting such experiments. Reciprocal transplantation of seedlings across environments are of course more easily conducted than common garden experiments, but they are still time consuming and costly; in addition species adapted to one soil type often will not survive when transplanted to other soil types.

## Conclusions

Although New Caledonian *Diospyros* are morphologically and ecologically diverse, they show little genetic divergence (based on DNA sequences and AFLP data). In this case of the endemic clade of New Caledonian *Diospyros*, AFLP data did not provide enough information to resolve phylogenetic relationships between the species, but it was sensitive enough for testing for the presence of genetic species boundaries. However, the AFLP results exhibit a good correlation with morphology-based species concepts. Further studies of this New Caledonian *Diospyros* group with deeper sampling of the genome using next generation sequencing methods are needed to get a clearer picture of the processes that formed this group.

## Methods

### Material

**Figure 5:**
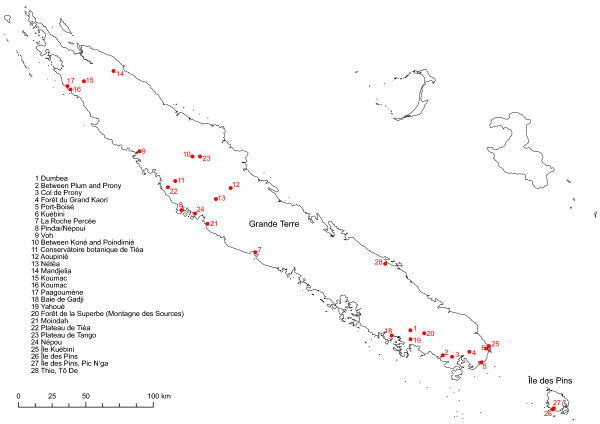
**Map with sampling localities.** Dots indicate sampling sites; the numbers associated with each dot refer to the list of sampling sites on this figure. Those numbers are used throughout the present study to characterize sampling sites.

Material from New Caledonian *Diospyros* species was collected on the main island (Grande Terre) and on a smaller island, Île des Pins. When possible, we collected five individuals per population. Collecting population samples from tropical trees/shrubs is not always easy because the trees can be tall (and leaves therefore out of reach) and individuals are often far from each other. Collecting ten individuals in an area of ten square meters also does not make much sense for a study like this because these individuals are probably offspring from the same mother plant. As the focus of the present study is on the phylogenetic relationships between the species and not on population genetics within species, the authors consider the small size of the samples we collected to be sufficient. For widespread species, we collected populations throughout their range. For distribution of sampling sites, see Figure 5. From samples where fertile material was available, a voucher was made with several duplicates sent to the herbaria at Noumea (NOU), University of Montpellier II (MPU) and the University of Vienna (WU). When sterile, one voucher per population was taken; this was compared to already existing collections in Noumea Herbarium (NOU) from the same location and referred to that species if similar. In total we included in the present study 231 individuals of New Caledonian *Diospyros* species, which correspond to 20 identified and one unidentified species (due to absence of diagnostic reproductive organs at the time of collection), giving 47 populations in total. Details of the 192 individuals (43 populations) for which we were able to get useable results are given in Table 3. Silica-gel-dried material was used for DNA extraction.

**Table 3:** Table of accessions; showing all individuals used in this study

**Taxon**	**Sample ID**	**Population**	**Sampling location**	**Voucher**
*D. calciphila* F.White	BT312-BT317	1	26, littoral forest	JM6650, JM6653
				(MPU, NOU, P)
*D. cherrieri* F.White	BT262, BT276-BT278	2	21, dry forest	NOU079551, WU062860
				NOU054492, NOU054008
*D. cherrieri*	BT293-BT297	3	24, dry forest	NOU079547
*D. erudita* F.White	BT259-BT261, BT273-BT275	4	21, dry forest	WU062855, NOU079545
				NOU079544, WU062870
				NOU054010, WU062856
				NOU054011, WU062857
*D. erudita*	BT280-BT285, BT287	5	22, dry forest	WU062858, Chambrey & Turner 20 (NOU)
*D. flavocarpa* (Vieill. ex P.Parm.) F.White	BT126-BT130	6	10, humid mountain forest	JM6625 (NOU)
*D. flavocarpa*	BT155, BT158-BT159	7	12, dense humid mountain forest	JM6632 (NOU)
*D. glans* F.White	BT020-BT022	8	1, forest near river	NOU053705, NOU030755, WU062846
*D. glans*	BT075	9	5, dense forest near road	NOU000819
*D. glans*	BT082, BT084, BT087, BT093-BT094	10	6, forest near river	NOU022860
*D. impolita* F.White	BT101-BT105	11	7, mesophyll forest near beach	NOU019538
*D. inexplorata* F.White	BT304, BT307-BT311	12	25, littoral forest	NOU005818
*D. labillardierei* F.White	BT121-BT125	13	10, river edge in mountain forest	JM6624 (NOU)
*D. labillardierei*	BT178-BT182	14	13, river edge	(NOU031346)
*D. minimifolia* F.White	BT134-BT135	15	11, dry forest	NOU019556
*D. minimifolia*	BT230-BT234	16	18, mesophyll forest near beach	NOU019554
*D. minimifolia*	BT263-BT264, BT266-267, BT269-BT270	17	21, dry forest	NOU079549, WU062872
				NOU054493
*D. pancheri* Kosterm.	BT029-BT031, BT035	18	2, forest near road	JM6619, JM6620 (NOU)
*D. pancheri*	BT076-BT079	19	5, dense forest near road	
*D. parviflora* (Schltr.) Bakh.	BT042	20	3, wet forest	
*D. parviflora*	BT059, BT062-BT063, BT068	21	4, wet dense forest	NOU006656
*D. parviflora*	BT080, BT085, BT089-BT090	22	6, forest near river	JM6622 (NOU)
*D. parviflora*	BT248-BT250, BT252-BT253	23	20, humid forest at low elevation	tree no. 23109
*D. parviflora*	BT289-BT292	24	23, mountain forest	NOU079550
*D. perplexa* F.White	BT147-BT151	25	11, forest near river	JM6630 (NOU)
*D. pustulata* F.White	BT111-BT114	26	8, dry forest	
*D. pustulata*	BT136-BT140	27	11, dry forest	JM6629 (NOU)
*D. pustulata*	BT257-BT258, BT265, BT268, BT271-BT272	28	21, dry forest	NOU079548, WU062871
				NOU053999
*D. revolutissima* F.White	BT116-BT120	29	9, maquis	NOU023189
*D. revolutissima*	BT218-BT222	30	17, maquis	JM6640 (NOU)
*D. tridentata* F.White	BT202-BT207	31	15, dry forest at low elevation	JM6639 (NOU)
*D. trisulca* F.White	BT185, BT192, BT197, BT199-BT201	32	14, mountain forest	NOU031344, JM6637 (NOU)
*D. umbrosa* F.White	BT061, BT065-BT066, BT071, BT073	33	4, wet dense forest	
*D. umbrosa*	BT170-BT171, BT175-BT177	34	13, dense humid forest	JM6635 (NOU)
*D. umbrosa*	BT246-BT247, BT251, BT254, BT256	35	20, humid forest at low elevation	NOU023234
*D. veillonii* F.White	BT224, BT226-BT229	36	18, mesophyll forest near beach	NOU019582
*D. vieillardii* (Hiern) Kosterm.	BT017, BT023-BT026	37	1, forest near river	JM6618 (NOU)
*D. vieillardii*	BT055, BT057-BT058	38	4, dry open forest	
*D. vieillardii*	BT088, BT091-BT092, BT098, BT100	39	6, forest near river	
*D. vieillardii*	BT215-BT217	40	16, maquis	NOU023242
*D. vieillardii*	BT324-BT325, BT328	41	28, forest near river	
*D. yahouensis* (Schltr.) Kosterm.	BT237-BT239	42	19, mesophyll forest	P00057340
*D.* sp. Pic N'ga	BT319, BT321-BT323	43	27, maquis	JM6065 (NOU)

The numbers of sampling localities are the same as in Figure 2. Voucher-Codes: JMXXXX: collection number J. Munzinger; Tree N° XXX: Tree of New Caledonian Plant Inventory and Permanent Plot Network (NC-PIPPN, [45]); NOUXXXXXXX: Herbarium accession number of Noumea herbarium (NOU); WUXXXXXX: Herbarium accession number of the Herbarium of the University Vienna (WU); P: Herbarium of the Natural History Museum Paris; MPU: Herbarium of the University of Montpellier II.

### DNA extraction

For DNA extraction, a modified sorbitol/high-salt CTAB method [46] was used (for details see [13]).

### AFLP

Preparation and amplification of fragments followed the protocol of Vos et al. [14] with some modifications. Restriction of genomic DNA with two restriction enzymes and ligation of double-stranded adaptors to the resulting restricted fragments were performed in one step in a thermal cycler (Veriti, AB, Life Technologies; 37°C for 2 h followed by a 30 min hold at 17°C). Reactions comprised 1.1 μL 10x T4 DNA ligase buffer (Promega), 1.1 μL 0.5 M NaCl, 0.55 μL BSA (1 mg/ mL; New England BioLabs), 50 μM MseI adaptors (genXpress), 5 μM EcoRI adaptors (genXpress), 1 U MseI restriction endonuclease (New England BioLabs), 5 U EcoRI restriction endonuclease (New England BioLabs), 1 U T4 DNA ligase (Promega), and 0.5 μg DNA and were made up to a total volume of 11 μL with water. Ligated DNA fragments were diluted 10-fold with sterile water. Preselective amplification reactions contained 1.14 μL 10x RedTaq PCR reaction buffer (Sigma), 0.2 U RedTaq DNA polymerase (Sigma), 0.22 μL dNTPs (10 mM; AB, Life Technologies), 0.58 μL preselective primer pairs (EcoRI-A and MseI-CT, each 5 μM; Sigma), and 2 μL diluted restriction-ligation product, and were brought with water to a total volume of 10 μL. Amplification was carried out in the same machine used for restriction-ligation with the following profile: 2 min at 72°C, 20 cycles of 10 sec denaturing at 94°C, 30 sec annealing at 56°C, 2 min extension at 72°C, and a final extension step for 30 min at 60°C. The preselective PCR products were diluted 10-fold with sterile water. Reactions for selective amplification contained 0.5 μL 10x RedTaq PCR reaction buffer (Sigma), 0.1 U RedTaq DNA polymerase (Sigma), 0.11 μL dNTPs (10 mM; AB, Life Technologies), 0.27 μL MseI-primer (5 μM; Sigma), 0.27 μL EcoRI-primer (1 μM; Sigma), and 1 μL diluted preselective amplification product and were brought to a total volume of 5 μL with water. They were carried out in a GeneAmp PCR System 9700 (AB, Life Technologies) with the following profile: 1 min at 94°C, 9 cycles of 1 sec at 94°C, 30 sec at 65-57°C (reducing the temperature at 1°C per cycle), 2 min at 72°C, 25 cycles of 1 sec at 94°C, 30 sec at 56°C, 2 min at 72°C and a final extension for 30 min at 60°C. The selective PCR products were purified using Sephadex G-50 Superfine (GE Healthcare Bio-Sciences) applied to a MultiScreen-HV 96-Well Plate (Millipore) in three steps of 200 μL each and settled at 750 g (1, 1 and 5 min, respectively). The same speed was used for centrifugation of the samples (5 μL of each selective PCR product), again for 5 min. Two microliters of the eluate were combined with 10 μL HiDi and 0.1 μL GeneScan 500 ROX (AB, Life Technologies) and denatured for 3 min at 95°C before running them on a capillary sequencer (3130xl Genetic Analyzer, AB, Life Technologies).

The selective primer pairs (6Fam-EcoRI-AGC/MseI-CTGA, Vic-EcoRI-ATG/MseI-CTCG and Ned-EcoRI-ATC/MseI-CTGA) were chosen because they generated clear and not too many bands (thus decreasing the risk of fragments co-migrating by chance), with sufficient variability in preliminary tests. Although the genome size of the New Caledonian *Diospyros* species (1C-value: 1.5 – 2.3 pg; [13]) is smaller than the mean 1C-value of eudicots (2.7 pg, [47]), we found the AFLP profiles generated with Msel primers with four selective bases much clearer than those obtained from primers with just three selective bases.

Reproducibility was checked by repeating ca. 80% of the samples. This high number of repetitions was necessary because of initial difficulties with fragment sizing.

### Scoring and phylogenetic analysis

Sizing and scoring of the data was performed with GeneMarker v2.2.0 (SoftGenetics). After pre-analysis using default settings, sizing profiles of all samples were checked and where necessary manually corrected. Most of these corrections concerned one of the following peaks of the size standard: 35 bp, 50 bp and 139 bp. These peaks were often not correctly recognized by the GeneMarker program. High-quality sizing-profiles (score > 90) were obtained for all samples. A panel of scorable fragments was established for each primer combination, and fragments between 65 – 510 bp were scored. The relative fluorescent unit (RFU) threshold was set at 40. Automatic scoring was conducted using Local Southern peak call, peak saturation, base line subtraction, spike removal, pull up correction, and a stutter peak filter of 5% (as described in [48]). The results were exported as presence/absence matrix. The outcome of the automatic scoring was manually checked and corrected for errors. These errors mostly concerned peaks for which shape was atypical. In total 486 samples corresponding to 231 individuals were scored. From 186 individuals replicate samples were performed (between two and five replicates per individual). The differences between the different samples (replicates) were counted and divided by the total number of phenotypic comparisons to get the error rate (calculated according to Bonin et al. [49]). After initial analysis (neighbour-joining, NJ) of the complete data set, replicates of samples and obviously failed samples were excluded from further analyses. As replicated samples of the corresponding individuals mostly clustered together, selection of samples from each individual for further analyses was random and not according to any pattern or protocol. For the final analyses we ended up with 192 individuals.

All three primer-combinations were combined in a single matrix and analysed together. Different distance measures were tested for their power to resolve relationships with our data set. Distance matrixes were calculated in PAUP* v4b10 ([50]; Nei-Li distance) and SplitsTree v4.12.6 ([51]; uncorrected P, Dice, corrected and uncorrected Hamming). Phylogenetic relationships based on previously mentioned distance matrices were reconstructed using SplitsTree v4.12.6 [51] to create unrooted NJ dendrograms. To assess robustness of branches NJ-bootstrap (NJ-BS) analyses were performed using SplitsTree v4.12.6 [51] and PAUP* v4b10 [50]. Bayesian inference (BI) was conducted with BEAST v1.7.5 [52], with two runs each 20 million generations, sampling every 1,000^th^ generation and removal of the first 30% of trees as burn in.

To visualise the pattern of genetic clustering of individuals and populations, we plotted principal coordinate analysis (PCO) using the R-package scatterplot3d [53] based on an individual Dice distance matrix, and respectively, on AMOVA-derived pair-wise *F*_*ST*_ distances calculated with Arlequin v3.5.1.2 [54]. To investigate further significant groupings of the included individuals we used the program Structure v2.3.3 [55,56] on the Bioportal computing cluster of the University Oslo [57]. We ran Structure for K = 1–23 with 10 replicates each and a model based on admixture and independent allelic frequencies, without taking into account information regarding sampling localities. Each run had 3 million iterations with 10% additional burn in. The calculation of deltaK (∆K; [27]) and preparation of the input file for Clumpp was done with Harvester [58]. Production of a combined file from the ten replicates of the best K was perfomed using Clumpp v1.1.2 [59] with the full search algorithm. The graphical representation of Structure results was prepared with Distruct v1.1 [60].

Both non-hierarchical and hierarchical analyses of molecular variance (AMOVA) and calculations of population statistics were conducted using Arlequin v3.5.1.2 [54]. For hierarchical AMOVAs groups have been defined based on different possible clusterings (Additional file 4) according to Structure results, taxonomy, distribution patterns and ecological traits.

### Availability of supporting data

AFLP presence/absence matrix and phylogenetic analyses are deposited in treeBASE under study 14798 (http://purl.org/phylo/treebase/phylows/study/TB2:S14798).

## Competing interests

The authors declare that they have no competing interests.

## Authors’ contributions

BT carried out the acquisition and analysis of the data, drafted the manuscript and assisted collecting the plant material. OP helped with data analysis. JM collect and identified the plant material and helped to interpret the results. Previous studies of SD helped to design this project. MWC helped to design this project and gave linguistic support to the manuscript. RS, designed and coordinated the study and helped to draft the manuscript. All authors read, commented, corrected and approved the final manuscript.

## Supplementary Material

Additional file 1 Structure**results of suboptimal K values (3, 6, 16 and 21) in comparison with K =2.** Delta K likelihoods are given for each K. Click here for file

Additional file 2 **Table showing the population statistics inferred from non-hierarchical AMOVA based on STRUCTURE results.** Populations marked bold differ in this analysis from the general population grouping given in Table 3. Click here for file

Additional file 3 **Figure of the neighbour joining dendrogram coloured according to soil type (colour of the branches) and water availability (colour of taxa names).** This dendrogram is the same as Figure 3A, but coloured according to ecological features. Click here for file

Additional file 4 **Table giving the details of the different AMOVAs conducted.** The numbers in the populations column are the same as given in Table 3, respectively, in Additional file 1 for the Structure based AMOVA. Click here for file

## References

[B1] Givnish TJ Millam KC Mast AR Paterson TB Theim TJ Hipp AL Henss JM Smith JF Wood KR Sytsma KJ Origin, adaptive radiation and diversification of the Hawaiian lobeliads (Asterales: Campanulaceae) Proc R Soc B 2009 13 407 146 10.1098/rspb.2008.1204 18854299 PMC2664350

[B2] Knope ML Morden CW Funk VA Fukami T Area and the rapid radiation of Hawaiian *Bidens* (Asteraceae) J Biogeogr 2012 13 1206 1216 10.1111/j.1365-2699.2012.02687.x

[B3] Mittermeier RA Gil PR Hoffmann M Pilgrim J Brooks T Mittermeier CG Lamoreux J da Fonseca GAB Hotspots revisited: Earth’s biologically richest and most endangered terrestrial ecoregions 2004 Mexico City: CEMEX

[B4] Myers N Mittermeier RA Mittermeier CG da Fonseca GAB Kent J Biodiversity hotspots for conservation priorities Nature 2000 13 853 858 10.1038/35002501 10706275

[B5] Morat P Jaffré T Tronchet F Munzinger J Pillon Y Veillon J-M Chalopin M Le référentiel taxonomique Florical et les caractéristiques de la flore vasculaire indigène de la Nouvelle-Calédonie Adansonia 2012 13 177 219

[B6] Lowry PP II Peng CF, Lowry PPII Diversity, endemism and extinction in the flora of New Caledonia: a review Rare, threatened, and endangered floras of Asia and the Pacific rim 1998 Taiwan: Institute of Botany, Taipei 181 206

[B7] Pelletier B Payri C Richer De Forges B Geology of the New Caledonia region and its implications for the study of the New Caledonian biodiversity Compendium of marine species from New Caledonia, Documents Scientifiques et Techniques II4 2006 New Caledonia: Institut de Recherche pour le Développement Nouméa 17 30

[B8] Maurizot P Vendé-Leclerc M New Caledonia geological map, scale 1/500000 Direction de l’Industrie, des Mines et de l’Energie - Service de la Géologie de Nouvelle-Calédonie, Bureau de Recherches Géologiques et Minières 2009

[B9] Pillon Y Munzinger J Amir H Lebrun M Ultramafic soils and species sorting in the flora of New Caledonia J Ecol 2010 13 1108 1116 10.1111/j.1365-2745.2010.01689.x

[B10] Jaffré T Rigault F Munzinger J Bonvallot J, Gay J-C, Habert E La végétation Atlas de la Nouvelle-Calédonie 2012 Nouméa: IRD Editions 77 80

[B11] Duangjai S Wallnöfer B Samuel R Munzinger J Chase MW Generic delimitation and relationships in Ebenaceae sensu lato: evidence from six plastid DNA regions Am J Bot 2006 13 1808 1827 10.3732/ajb.93.12.1808 21642127

[B12] Duangjai S Samuel R Munzinger J Forest F Wallnöfer B Barfuss MHJ Fischer G Chase MW A multi-locus plastid phylogenetic analysis of the pantropical genus *Diospyros* (Ebenaceae), with an emphasis on the radiation and biogeographic origins of the New Caledonian endemic species Mol Phylogenet Evol 2009 13 602 620 10.1016/j.ympev.2009.04.021 19427384

[B13] Turner B Munzinger J Duangjai S Temsch EM Stockenhuber R Barfuss MHJ Chase MW Samuel R Molecular phylogenetic of New Caledonian *Diospyros* (Ebenaceae) using plastid and nuclear markers Mol Phylogenet Evol 2013 13 740 763 10.1016/j.ympev.2013.07.002 23850609 PMC3913082

[B14] Vos P Hogers R Bleeker M Reijans M Van de Lee T Hornes M Frijters A Pot J Peleman J Kuiper M Zabeau M AFLP: a new technique for DNA fingerprinting Nucleic Acids Res 1995 13 4407 4414 10.1093/nar/23.21.4407 7501463 PMC307397

[B15] Tremetsberger K Stuessy TF Kadlec G Urtubey E Baeza CM Beck SG Valdebenito HA Ruas CF Matzenbacher NI AFLP phylogeny of South American species of *Hypochaeris* (Asteraceae, Lactuceae) Syst Bot 2006 13 610 626 10.1600/036364406778388520

[B16] Koopman WJM Zevenbergen MJ van den Berg RG Species relationships in *Lactuca s.l.* (Lactuceae, Asteraceae) inferred from AFLP fingerprints Am J Bot 2001 13 1881 1887 10.2307/3558364 21669621

[B17] Richardson JE Fay MF Cronk QCB Chase MW Species delimitation and the origin of populations in island representatives of *Phylica* (Rhamnaceae) Evolution 2003 13 816 827 12778551 10.1111/j.0014-3820.2003.tb00293.x

[B18] Despré L Giells L Redoutet B Taberlet P Using AFLP to resolve phylogenetic relationships in a morphologically diversified plant species complex when nuclear and chloroplast sequences fail to reveal variability Mol Phylogenet Evol 2003 13 185 196 10.1016/S1055-7903(02)00445-1 12695084

[B19] Paun O Schönswetter P Winkler M Tribsch A IntraBioDiv Consortium Historical divergence vs. contemporary gene flow: evolutionary history of the calcicole Ranunculus alpestris group (Ranunculaceae) in the European Alps and the Carpathians Mol Ecol 2008 13 4263 4275 10.1111/j.1365-294X.2008.03908.x 19378404 PMC2988486

[B20] Schulte K Silvestro D Kiehlmann E Vesely S Novoa P Zizka G Detection of recent hybridization between sympatric Chilean Puya species (Bromeliaceae) using AFLP markers and reconstruction of complex relationships Mol Phylogenet Evol 2010 13 1105 1119 10.1016/j.ympev.2010.09.001 20832496

[B21] Jabaily RS Sytsma KJ Historical biogeography and life-history evolution of Andean Puya (Bromeliaceae) Bot J Linn Soc 2012 13 201 224

[B22] Gaudeul M Rouhan G Gardner MF Hollingsworth PM AFLP markers provide insights into the evolutionary relationships and diversification of New Caledonian Araucaria species (Araucariaceae) Am J Bot 2012 13 68 81 10.3732/ajb.1100321 22184275

[B23] Koopman WJM Phylogenetic signal in AFLP data sets Syst Biol 2005 13 197 217 10.1080/10635150590924181 16012092

[B24] Degnan JH Rosenberg NA Discordance of species trees with their most likely gene trees PLoS Genet 2006 13 762 768 10.1371/journal.pgen.0020068 PMC1464820 16733550

[B25] Meudt HM Clarke AC Almost forgotten or latest practice? AFLP applications, analyses and advances Trends Plant Sci 2007 13 106 117 10.1016/j.tplants.2007.02.001 17303467

[B26] Bussell JD Waycott M Chappill JA Arbitrarily amplified DNA markers as characters for phylogenetic inference Perspect Plant Ecol, Evol Systematics 2005 13 3 26 10.1016/j.ppees.2004.07.001

[B27] Evanno G Regnaut S Goudet J Detecting the number of clusters of individuals using the software Structure: a simulation study Mol Ecol 2005 13 2611 2620 10.1111/j.1365-294X.2005.02553.x 15969739

[B28] Vigouroux Y Glaubitz JC Matsouka Y Goddman MM Sánchez GJ Doebley J Population structure and genetic diversity of New World maize races assessed by DNA microsatellites Am J Bot 2008 13 1240 1253 21632329 10.3732/ajb.0800097

[B29] Glor RE Phylogenetic insights on adaptive radiation Ann Rev Ecol, Evol Systematics 2010 13 251 270 10.1146/annurev.ecolsys.39.110707.173447

[B30] Lerner HRL Meyer M James HF Hofreiter M Fleischer RC Multilocus resolution of phylogeny and timescale in the extant adaptive radiation of Hawaiian honeycreepers Curr Biol 2011 13 1838 1844 10.1016/j.cub.2011.09.039 22018543

[B31] White F Flore de la Nouvelle-Calédonie et Dépendances. 19. Ébénacées 1993 Paris: Muséum National d’Histoire Naturelle

[B32] Kapralov MV Votintseva AA Filatov DA Molecular adaptation during a rapid adaptive radiation Mol Biol Evol 2013 13 1051 1059 10.1093/molbev/mst013 23355532 PMC3670742

[B33] Schmidt JM Good RT Appleton B Sherrard J Raymant GC Copy number variation and transposable elements feature in recent ongoing adaptation at the Cyp6g1 locus PLoS Genet 2010 13 e1000998 10.1371/journal.pgen.1000998 20585622 PMC2891717

[B34] Pintaud J-C Tanguy J Puig H Chorology of New Caledonian palms and possible evidence of Pleistocene rain forest refugia C R Acad Sci 2011 13 453 463 10.1016/s0764-4469(01)01312-9 11411288

[B35] Pillon Y Hopkins HC Munzinger J Amir H Chase MW Cryptic species, gene recombination and hybridization in the genus Spiraeanthemum (Cunoniaceae) from New Caledonia Bot J Linn Soc 2009 13 137 152 10.1111/j.1095-8339.2009.00997.x

[B36] Poncet V Munoz F Munzinger J Pillon Y Gomez C Couderc M Tranchant-Dubreuil C Hamon S de Kochko A Phylogeography and niche modelling of the relict plant Amborella trichopoda (Amborellaceae) reveal multiple Pleistocene refugia in New Caledonia Mol Ecol 2013 doi:10.1111/mec.12554 10.1111/mec.12554 24118476

[B37] Bennett KD Milankovitch cycles and their effects on species in ecological and evolutionary time Paleobiology 1990 13 11 21

[B38] Kane NC King MG Barker MS Raduski A Karrenberg S Yatabe Y Knapp SJ Rieseberg LH Comparative genomic and population genetic analyses indicate highly porous genomes and high levels of gene flow between divergent Helianthus species Evolution 2009 13 2061 2075 10.1111/j.1558-5646.2009.00703.x 19473382 PMC2731706

[B39] Meudt HM Lockhart PJ Bryant D Species delimitation and phylogeny of a New Zealand plant species radiation BMC Evol Biol 2009 13 111 10.1186/1471-2148-9-111 19457251 PMC2700801

[B40] Pillon Y Munzinger J Amir H Hopkins HC Chase MW Reticulate evolution on a mosaic of soils: diversification of the New Caledonian endemic genus Codia (Cunoniaceae) Mol Ecol 2009 13 2263 2275 10.1111/j.1365-294X.2009.04178.x 19389179

[B41] Murienne J Guilbert E Grandcolas P Species diversity in the New Caledonian endemic genera Cephalidiosus and Nobarnus (Insecta: Heteroptera: Tingidae), an approach using phylogeny and species distribution modelling Biol J Linn Soc 2009 13 177 184 10.1111/j.1095-8312.2008.01184.x

[B42] Givnish TJ Montgomery RA Goldstein G Adaptive radiation of photosynthetic physiology in the Hawaiian lobeliads: light regimes, static light responses, and whole-plant compensation points Am J Bot 2004 13 228 246 10.3732/ajb.91.2.228 21653379

[B43] Baldwin BG Sanderson MJ Age and rate of diversification of the Hawaiian silversword alliance (Compositae) Proc Natl Acad Sci USA 1998 13 9402 9406 10.1073/pnas.95.16.9402 9689092 PMC21350

[B44] Barraclough TG What can phylogenetics tell us about speciation in the Cape flora? Divers Distrib 2006 13 21 26 10.1111/j.1366-9516.2006.00208.x

[B45] Ibanez T Munzinger J Dagostini G Hequet V Rigault F Jaffré T Birnbaum P Structural and floristic diversity of mixed tropical rainforest in New Caledonia: New data from the New Caledonian Plant Inventory and Permanent Plot Network (NC-PIPPN) Appl Veg Scidoi:10.1111/avsc.1270

[B46] Tel-Zur N Abbo S Myslabodski D Mizrahi Y Modified CTAB procedure for DNA isolation from epiphytic cacti of genera Hylocereus and Selenicereus (Cactaceae) Plant Mol Biol Report 1999 13 249 254 10.1023/A:1007656315275

[B47] Bennett MD Leitch IJ Angiosperm DNA *C*-values database (release 8.0, Dec. 2012) http://www.kew.org/cvalues/

[B48] Safer S Tremetsberger K Guo Y-P Kohl G Samuel MR Stuessy TF Stuppner H Phylogenetic relationships in the genus Leontopodium (Asteraceae: Gnaphalieae) based on AFLP data Bot J Linn Soc 2011 13 364 377 10.1111/j.1095-8339.2011.01117.x 23258943 PMC3524420

[B49] Bonin A Bellemain E Bronken Eidesen P Pompanon F Brochmann C Taberlet P How to track and assess genotyping errors in population genetic studies Mol Ecol 2004 13 3261 3273 10.1111/j.1365-294X.2004.02346.x 15487987

[B50] Swofford DL PAUP*. Phylogenetic analysis using parsimony (*and other methods). Version 4 2003 Sunderland, Massachusetts: Sinauer Associates

[B51] Huson DH Bryant D Application of phylogenetic networks in evolutionary studies Mol Biol Evol 2006 13 254 267 16221896 10.1093/molbev/msj030

[B52] Drummond AJ Suchard MA Xie D Rambaut A Bayesian phylogenetics with BEAUti and the BEAST 1.7 Mol Biol Evol 2012 13 1969 1973 10.1093/molbev/mss075 22367748 PMC3408070

[B53] Ligges U Mächler M Scatterplot3d - an R package for visualizing multivariate data J Stat Softw 2003 13 1 20

[B54] Excoffier L Laval G Schneider S Arlequin (version 3.0): an integrated software package for population genetics data analysis Evol Bioinformatics Online 2005 13 47 50 PMC2658868 19325852

[B55] Pritchard JK Stephens M Donnely P Inference of population structure using multilocus genotype data Genetics 2000 13 945 959 10835412 10.1093/genetics/155.2.945 PMC1461096

[B56] Hubisz MJ Falush D Stephens M Pritchard JK Inferring weak population structure with the assistance of sample group information Mol Ecol Resour 2009 13 1322 1332 10.1111/j.1755-0998.2009.02591.x 21564903 PMC3518025

[B57] Lifeportal http://www.uio.no/english/services/it/research/hpc/lifeportal/

[B58] Earl DA Von Holdt BM Structure Harvester: a website and program for visualizing Structure output and implementing the Evanno method Conserv Genet Resour 2012 13 359 361 10.1007/s12686-011-9548-7

[B59] Jakobsson M Rosenberg NA CLUMPP: a cluster matching and permutation program for dealing with label switching and multimodality in analysis of population structure Bioinformatics 2007 13 1801 1806 10.1093/bioinformatics/btm233 17485429

[B60] Rosenberg NA Distruct: a program for the graphical display of population structure Mol Ecol Notes 2004 13 137 138

