# Supplementary material for: Analyses of amplified fragment length polymorphisms (AFLP) indicate rapid radiation of Diospyros species (Ebenaceae) endemic to New Caledonia
Source: BMC Evol Biol. 2013 Dec 12;13:269. doi: 10.1186/1471-2148-13-269 (PMC3881503; doi:10.1186/1471-2148-13-269)
Supplement: Additional file 1 — Structureresults of suboptimal K values (3, 6, 16 and 21) in comparison with K =2. Delta K likelihoods are given for each K. [file 1471-2148-13-269-S1.pdf]

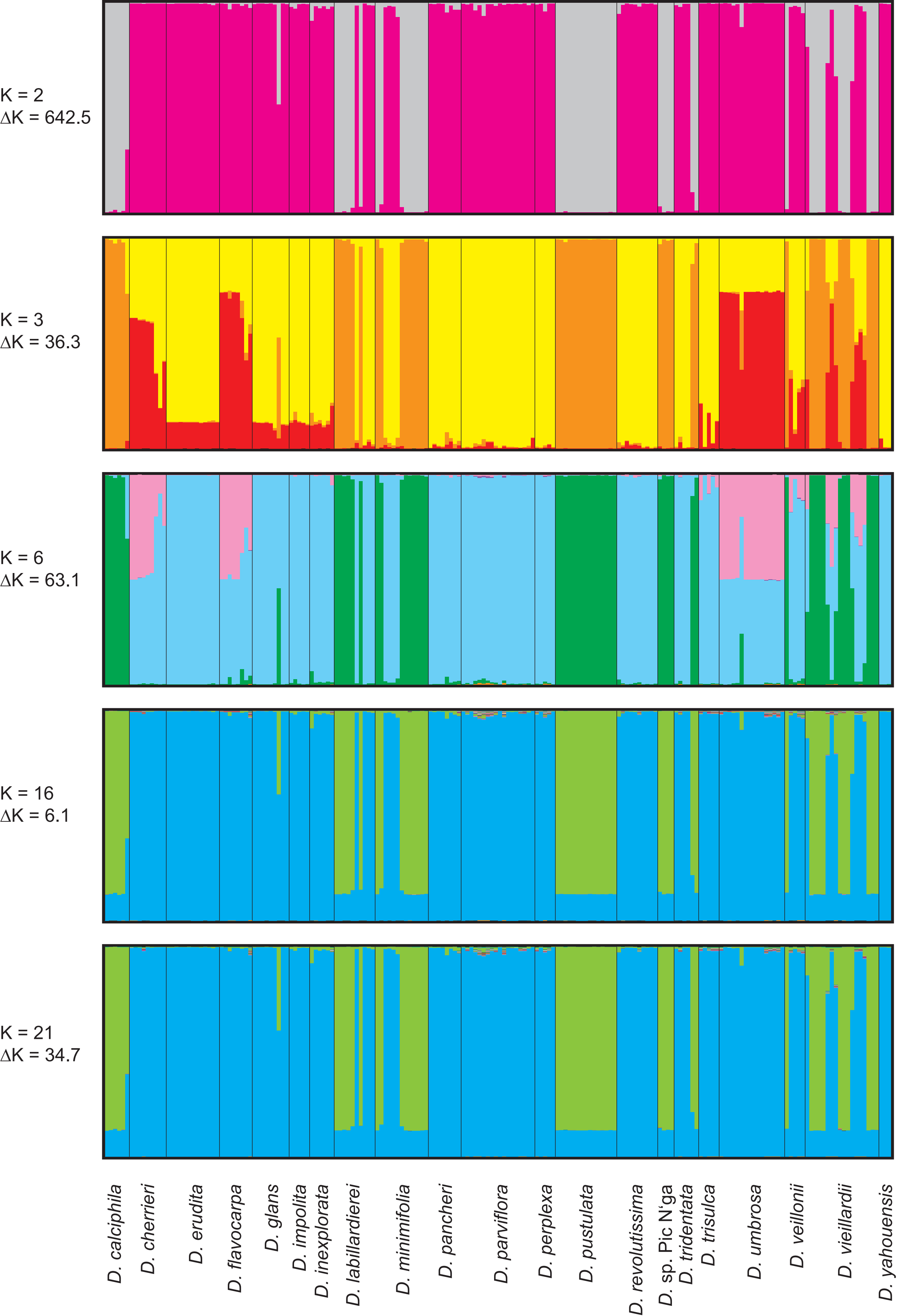
K = 2
ΔK = 642.5
K = 3
ΔK = 36.3
K = 6
ΔK = 63.1
K = 16
ΔK = 6.1
K = 21
ΔK = 34.7
D. calciphila
D. cherrieri
D. erudita
D. flavocarpa
D. glans
D. impolita
D. inexplorata
D. labillardierei
D. minimifolia
D. pancheri
D. parviflora
D. perplexa
D. pustulata
D. revolutissima
D. sp. Pic N'ga
D. tridentata
D. trisulca
D. umbrosa
D. veillonii
D. vieillardii
D. yahouensis
