# Supplementary material for: Analyses of amplified fragment length polymorphisms (AFLP) indicate rapid radiation of Diospyros species (Ebenaceae) endemic to New Caledonia
Source: BMC Evol Biol. 2013 Dec 12;13:269. doi: 10.1186/1471-2148-13-269 (PMC3881503; doi:10.1186/1471-2148-13-269)
Supplement: Additional file 2 — Table showing the population statistics inferred from non-hierarchical AMOVA based on STRUCTURE results. Populations marked bold differ in this analysis from the general population grouping given in Table 3. [file 1471-2148-13-269-S2.docx]

| Population | Taxon | Sample ID | Number of individuals | Number of polymorphic sites | Pairwise difference | Average gene diversity over loci | Pairwise difference no admixed accessions | Average gene diversity over loci no admixed accession |
| --- | --- | --- | --- | --- | --- | --- | --- | --- |
| 01 | *D. calciphila* | BT312-BT317 | 6 | 123 | 52.3 | 0.067 | 51.4 | 0.066 |
| 02 | *D. cherrieri* | BT262, BT276-BT278 | 4 | 52 | 29.5 | 0.037 |  |  |
| 03 | *D. cherrieri* | BT293-BT297 | 5 | 69 | 33.4 | 0.042 |  |  |
| 04 | *D. erudita* | BT259-BT261, BT273-BT275 | 6 | 48 | 22.1 | 0.028 |  |  |
| 05 | *D. erudita* | BT280-BT285, BT287 | 7 | 110 | 44.5 | 0.056 |  |  |
| 06 | *D. flavocarpa* | BT126-BT130 | 5 | 66 | 32.6 | 0.041 |  |  |
| 07 | *D. flavocarpa* | BT155, BT158-BT159 | 3 | 109 | 72.7 | 0.092 |  |  |
| 08 | *D. glans* | BT020-BT022 | 3 | 45 | 30.0 | 0.038 |  |  |
| 09 | *D. glans* | BT075 | 1 | - | - | - |  |  |
| 10 | *D. glans* | BT082, BT084, BT087, BT093-BT094 | 5 | 121 | 55.8 | 0.070 | 51.5 | 0.065 |
| 11 | *D. impolita* | BT101-BT105 | 5 | 105 | 50.2 | 0.063 |  |  |
| 12 | *D. inexplorata* | BT304, BT307-BT311 | 6 | 137 | 60.5 | 0.076 |  |  |
| **13** | ***D. labillardierei*** | **BT121-BT125, BT179** | **6** | **91** | **39.4** | **0.050** |  |  |
| **14** | ***D. labillardierei*** | **BT178, BT180-BT182** | **4** | **91** | **48.7** | **0.061** |  |  |
| **15** | ***D. minimifolia*** | **BT134-BT135, BT234** | **3** | **78** | **52.0** | **0.066** |  |  |
| **16** | ***D. minimifolia*** | **BT230-BT233** | **4** | **87** | **46.7** | **0.059** |  |  |
| 17 | *D. minimifolia* | BT263-BT264, BT266-267, BT269-BT270 | 6 | 166 | 71.5 | 0.090 |  |  |
| 18 | *D. pancheri* | BT029-BT031, BT035 | 4 | 118 | 64.0 | 0.081 |  |  |
| 19 | *D. pancheri* | BT076-BT079 | 4 | 131 | 71.5 | 0.090 |  |  |
| 20 | *D. parviflora* | BT042 | 1 | - | - | - |  |  |
| 21 | *D. parviflora* | BT059, BT062-BT063, BT068 | 4 | 154 | 85.0 | 0.107 |  |  |
| 22 | *D. parviflora* | BT080, BT085, BT089-BT090 | 4 | 176 | 94.8 | 0.120 |  |  |
| 23 | *D. parviflora* | BT248-BT250, BT252-BT253 | 5 | 170 | 79.6 | 0.100 |  |  |
| 24 | *D. parviflora* | BT289-BT292 | 4 | 125 | 68.2 | 0.086 |  |  |
| 25 | *D. perplexa* | BT147-BT151 | 5 | 161 | 75.4 | 0.095 |  |  |
| 26 | *D. pustulata* | BT111-BT114 | 4 | 82 | 43.5 | 0.055 |  |  |
| 27 | *D. pustulata* | BT136-BT140 | 5 | 79 | 37.4 | 0.047 |  |  |
| 28 | *D. pustulata* | BT257-BT258, BT265, BT268, BT271-BT272 | 6 | 105 | 45.2 | 0.057 |  |  |
| 29 | *D. revolutissima* | BT116-BT120 | 5 | 143 | 67.2 | 0.085 |  |  |
| 30 | *D. revolutissima* | BT218-BT222 | 5 | 124 | 59.2 | 0.075 |  |  |
| **31a** | ***D. tridentata*** | **BT202-BT205** | **4** | **110** | **58.7** | **0.074** |  |  |
| **31b** | ***D. tridentata*** | **BT206-BT207** | **2** | **45** | **45.0** | **0.057** |  |  |
| 32 | *D. trisulca* | BT185, BT192, BT197, BT199-BT201 | 5 | 155 | 75.4 | 0.095 |  |  |
| 33 | *D. umbrosa* | BT061, BT065-BT066, BT071, BT073 | 5 | 104 | 46.6 | 0.059 |  |  |
| 34 | *D. umbrosa* | BT170-BT171, BT175-BT177 | 5 | 59 | 28.4 | 0.036 |  |  |
| 35 | *D. umbrosa* | BT246-BT247, BT251, BT254, BT256 | 5 | 149 | 70.2 | 0.088 |  |  |
| **36a** | ***D. veillonii*** | **BT224** | **1** | **-** | **-** | **-** |  |  |
| **36b** | ***D. veillonii*** | **BT226-BT229** | **4** | **106** | **57.2** | **0.072** |  |  |
| **37a** | ***D. vieillardii*** | **BT017, BT088, BT100** | **3** | **86** | **57.3** | **0.072** | **-** | **-** |
| **37b** | ***D. vieillardii*** | **BT023-BT026** | **4** | **90** | **48.8** | **0.062** |  |  |
| 38 | *D. vieillardii* | BT055, BT057-BT058 | 3 | 120 | 80.0 | 0.101 | - | - |
| **39** | ***D. vieillardii*** | **BT091-BT092, BT098** | **3** | **108** | **72.0** | **0.091** |  |  |
| 40 | *D. vieillardii* | BT215-BT217 | 3 | 82 | 54.7 | 0.069 |  |  |
| 41 | *D. vieillardii* | BT324-BT325, BT328 | 3 | 74 | 49.3 | 0.062 |  |  |
| 42 | *D. yahouensis* | BT237-BT239 | 3 | 72 | 48.0 | 0.061 |  |  |
| 43 | *D.* sp. Pic N'ga | BT319, BT321-BT323 | 4 | 110 | 60.3 | 0.076 |  |  |
