# Supplementary figures and images for: Analyses of amplified fragment length polymorphisms (AFLP) indicate rapid radiation of Diospyros species (Ebenaceae) endemic to New Caledonia
Source: BMC Evol Biol. 2013 Dec 12;13:269. doi: 10.1186/1471-2148-13-269 (PMC3881503; doi:10.1186/1471-2148-13-269)

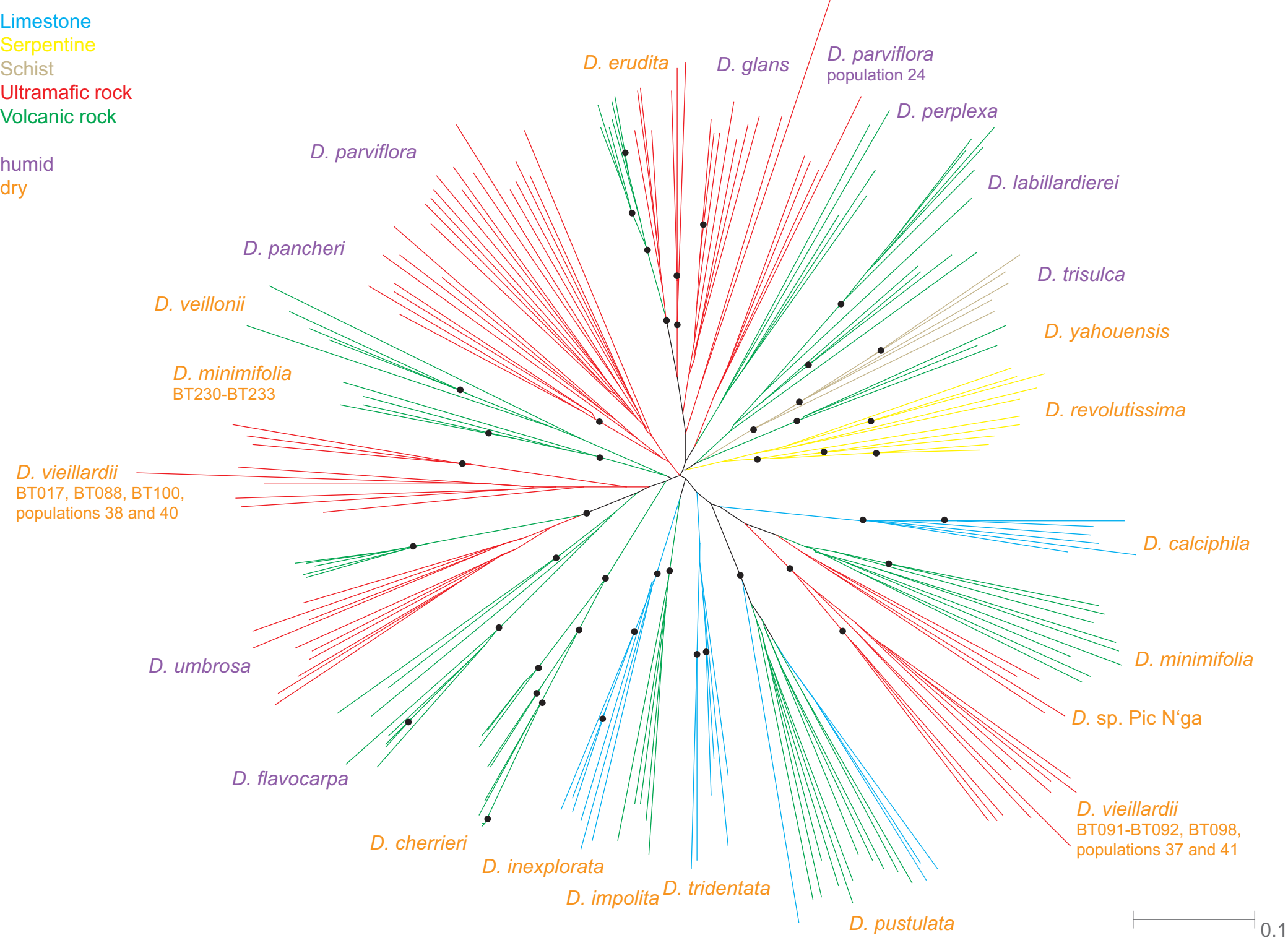

Supplement: Additional file 3 — Figure of the neighbour joining dendrogram coloured according to soil type (colour of the branches) and water availability (colour of taxa names). This dendrogram is the same as Figure 3A, but coloured according to ecological features. [file 1471-2148-13-269-S3.pdf]
