# Supplementary material for: Analyses of amplified fragment length polymorphisms (AFLP) indicate rapid radiation of Diospyros species (Ebenaceae) endemic to New Caledonia
Source: BMC Evol Biol. 2013 Dec 12;13:269. doi: 10.1186/1471-2148-13-269 (PMC3881503; doi:10.1186/1471-2148-13-269)
Supplement: Additional file 4 — Table giving the details of the different AMOVAs conducted. The numbers in the populations column are the same as given in Table 3, respectively, in Additional file 1 for the Structure based AMOVA. [file 1471-2148-13-269-S4.docx]

| Analysis | No of groups | Groups | Populations within groups |
| --- | --- | --- | --- |
| non-hierarchical | 1 | all | 01 - 43 |
| species wise | 21 | calciphila | 01 |
|  |  | cherrieri | 02, 03 |
|  |  | erudita | 04, 05 |
|  |  | flavocarpa | 06, 07 |
|  |  | glans | 08, 09, 10 |
|  |  | impolita | 11 |
|  |  | inexplorata | 12 |
|  |  | labillardierei | 13, 14 |
|  |  | minimifolia | 15, 16, 17 |
|  |  | pancheri | 18, 19 |
|  |  | parviflora | 20, 21, 22, 23, 24 |
|  |  | perplexa | 25 |
|  |  | pustulata | 26, 27, 28 |
|  |  | revolutissima | 29, 30 |
|  |  | tridentata | 31 |
|  |  | trisulca | 32 |
|  |  | umbrosa | 33, 34, 35 |
|  |  | veillonii | 36 |
|  |  | vieillardii | 37, 38, 39, 40, 41 |
|  |  | yahouensis | 42 |
|  |  | sp Pic N’ga | 43 |
| Structure | 2 | White | 01, 13, 15, 17, 26 - 28, 31b, 36a, 37b, 39, 41, 43 |
|  |  | Grey | 02 - 12, 14, 16, 18 - 25, 29 – 30, 31a, 32 – 34, 36b, 37a, 38, 40, 42 |
| geographic | 3 | north | 30 - 32, 40 |
|  |  | middle | 02 - 07, 11, 13 - 15, 17, 24 - 29, 34 |
|  |  | south | 01, 08 - 10, 12, 16, 18 - 23, 33, 35 - 39, 41 - 43 |
| water | 2 | dry | 01 - 05, 11, 12, 15 - 17, 26 - 31, 36, 38, 40, 42, 43 |
|  |  | humid | 06 - 10, 13, 14, 18 - 25, 32 - 35, 37, 39, 41 |
| soil | 5 | ultramafic | 05, 08 - 10, 18 - 24, 33, 35, 37 - 39, 41, 43 |
|  |  | limestone | 01, 12, 26, 31 |
|  |  | volcanic | 02 - 04, 06, 07, 11, 13 - 17, 25, 27, 28, 34, 36, 42 |
|  |  | serpentine | 29, 30, 40 |
|  |  | schist | 32 |
